# Reinforcement and Punishment Shape the Learning Dynamics in fMRI Neurofeedback

**DOI:** 10.3389/fnhum.2020.00304

**Published:** 2020-07-24

**Authors:** Manfred Klöbl, Paul Michenthaler, Godber Mathis Godbersen, Simon Robinson, Andreas Hahn, Rupert Lanzenberger

**Affiliations:** ^1^Department of Psychiatry and Psychotherapy, Medical University of Vienna, Vienna, Austria; ^2^Department of Biomedical Imaging and Image-guided Therapy, Medical University of Vienna, Vienna, Austria; ^3^Centre for Advanced Imaging, University of Queensland, Brisbane, QLD, Australia; ^4^Department of Neurology, Medical University of Graz, Graz, Austria

**Keywords:** neurofeedback, punishment, reinforcement (psychology), emotions, magnetic resonance imaging

## Abstract

**Introduction:**

Neurofeedback (NF) using real-time functional magnetic resonance imaging (fMRI) has proven to be a valuable neuroscientific tool for probing cognition and promising therapeutic approach for several psychiatric disorders. Even though learning constitutes an elementary aspect of NF, the question whether certain training schemes might positively influence its dynamics has largely been neglected.

**Methods:**

To address this issue, participants were trained to exert control on their subgenual anterior cingulate cortex (sgACC) blood-oxygenation-level-dependent signal, receiving either exclusively positive reinforcement (PR, “positive feedback”) or also positive punishment (PP, “negative feedback”). The temporal dynamics of the learning process were investigated by individually modeling the feedback periods and trends, offering the possibility to assess activation changes within and across blocks, runs and sessions.

**Results:**

The results show faster initial learning of the PR + PP group by significantly lower deactivations of the sgACC in the first session and stronger regulation trends during the first runs. Independent of the group, significant control over the sgACC could further be shown with but not without feedback.

**Conclusion:**

The beneficial effect of PP is supported by previous findings of multiple research domains suggesting that error avoidance represents an important motivational factor of learning, which complements the reward spectrum. This hypothesis warrants further investigation with respect to NF, as it could offer a way to generally facilitate the process of gaining volitional control over brain activity.

## Introduction

Neurofeedback (NF) utilizing functional Magnetic Resonance Imaging (fMRI) is a method for learning to gain control over the activation of almost any region of the brain. This is an important advantage compared to the traditional NF approaches using electroencephalography (EEG) or near-infrared spectroscopy, which are methodically limited to mostly large cortical regions. fMRI NF has been shown to be a promising therapeutic option for the treatment of diverse psychiatric and neurological conditions (Marzbani et al., 2016). Furthermore, it can be used for individual training and improvement of mental abilities (Yamashita et al., 2017) and it offers a tool to probe and potentially manipulate cognition *per se* (Sorger et al., 2018).

Many fMRI NF studies use simple block designs for training where subjects apply a certain strategy (regulation block) with in-between pauses (baseline). Runs without feedback (transfer runs; TRs), in which the subjects still have to apply the strategies learned, are often used to test for generalizability of the training successes. Even though the feedback provided to the subjects is often a continuous graphical representation of the target region’s activation (Sokunbi, 2017), the offline analyses of the recorded data usually follow more static approaches. The most widespread approach is the general linear model (GLM) for the analysis of whole-brain neuroimaging data and the reduction of training blocks, runs or sessions to single values per subject for subsequent statistical testing. Within the scope of this work, these terms are used as follows: training block: the continuous period during which the signal should be influenced; run: a single fMRI recording; session: everything that happens between the participant entering and leaving the MRI scanner.

Because learning is a dynamic process and it requires time to apply the regulation strategy [e.g., recall autobiographic memories to evoke certain emotions (Zotev et al., 2011) or imagine specific actions in sufficient intensity (Scharnowski et al., 2015)], systematic changes within a training block but also across runs or sessions can be expected (Hamilton et al., 2011). Although changes over sessions or between TRs are often used to show learning successes in NF, investigating the changes within blocks or feedback runs could give further insight into the perception or experiences of the individual subjects. Given recent findings on the importance of several psychological factors on the success of NF and also in the light of clinical trials, the additional information of single regulation blocks could be used to optimize the treatment protocol and uncover confounding effects like unnecessary long sessions and diminishing motivation or performance of patients (Kadosh and Staunton, 2019).

The type of feedback provided to the subjects also needs to be taken into account: A graphical presentation related to the goal of the NF training or possible strategies can, e.g., lead to improved regulation results compared to a neutral depiction (Mathiak et al., 2015). On top of this, the valence of the feedback signal has to be considered: Feedback which is provided only for volitional activation changes in the desired direction (positive reinforcement, PR) does not capture the full range of effects, even though the experience might be less frustrating for the subject. In contrast, feedback spanning the whole range of possible values [i.e., also including positive punishment, PP (Fernández et al., 2008)] provides additional information and might thereby foster faster learning.

Therefore, this study investigated the influence of the feedback type on the regulation and learning dynamics by comparing two groups, one receiving only PR feedback and the other PR + PP. The subgenual anterior cingulate cortex (sgACC) was selected as target since it represents an important emotion-related brain region typically affected in mood disorders such as major depression (Lanzenberger et al., 2013; Hoflich et al., 2017). Moreover, it was shown to be an effective and specific treatment target for deep brain stimulation (Drevets et al., 2008) and volitionally controllable using fMRI NF (Hamilton et al., 2011).

## Materials and Methods

This study was conducted in accordance with the Declaration of Helsinki and the good scientific practice guidelines of the Medical University of Vienna and approved by its ethics committee (ethics committee number 1937/2016).

### Subjects

Healthy volunteers were recruited via postings on message boards at the General Hospital of Vienna and nearby supermarkets, and from a database of potential subjects kept at the Department of Psychiatry and Psychotherapy. The inclusion criteria comprised an age of 18–35 years, right-handedness, general physical and mental health assessed via a thorough anamnesis and the axis I and II structural clinical interview according to the Diagnostic and Statistical Manual of Mental Disorders, version 4 (SCID I and II for DSM-IV), and signing of the informed consent form. Subjects were excluded in case of any MR incompatibilities or pregnancy, later discoveries of major internal, neurological or psychiatric illnesses, current substance abuse, when having smoked within 2 h before an MRI session or tried to cheat during the NF training (e.g., by changing their breathing pattern). The PR and PR + PP group were matched for sex, and mean and standard deviation of age. Thirteen volunteers were enrolled in the study (recruiting was continued until both groups contained at least five subjects with two successful NF sessions).

### Study Design

Each subject participated in two identical NF sessions, which were separated by 1–12 days. They were given a detailed instruction sheet with explanations on the different aspects of the study, which were also discussed with the experimenter before the first session. The subjects were not informed about the two different feedback schemes since group assignment would inevitably be revealed in the first NF run.

Each session started with a short questionnaire [the German short form of the Positive And Negative Affect Schedule (PANAS-SF) (Kercher, 1992; Breyer and Bluemke, 2016)], in which the subjects had to rate five positive and five negative adjectives depending on how much they currently applied to them on a 5-point (0–4) Likert scale.

The following measurements were performed in the order: (1) pre-NF resting-state (RS), (2) functional localizer (FL), (3) pre-NF TR, (4–6) three NF runs, (7) post-NF RS, (8) post-NF TR. An additional T1-weighted anatomical scan was acquired at the end of the first session as structural reference if functional images indicated any abnormality. The RS and anatomical data are not presented here.

After the measurements, the subjects were given the same questionnaire again and asked to rate their own performance using a visual analog scale (VAS) from 0 to 100%. Each session was concluded with a short interview by the experimenter regarding the strategies used and personal experiences. During the final examination, the subjects were also asked how they arrived at their most successful regulation strategies and whether they had preferred the feedback to be limited to the positive (PR + PP group) or to also include the negative range (PR-only group).

### Functional Localizer

A functional localizer was run in each session to allow delineation of the sgACC (Hamilton et al., 2011). This comprised five blocks of images with strongly negative valence from the validated EmoPics dataset (Wessa et al., 2010) alternated with five block of commands to press and hold a button on an MR-compatible keyboard (Current Designs, Philadelphia, PA, United States) as active baseline condition. The pictures were selected randomly without replacement to reduce a possible scene-dependent bias. In every block, either 3 images were shown for 6 s each, or 6 commands for 3 s. Overall, the FL took 3 min. Delineation of the target region of interest (ROI) was performed manually in Turbo BrainVoyager 4.0 beta (TBV) by selecting the active voxels inside the sgACC on the underlying functional reference image after varying the significance threshold until a reasonable coverage was achieved.

### Neurofeedback Presentation

**FIGURE 1:**
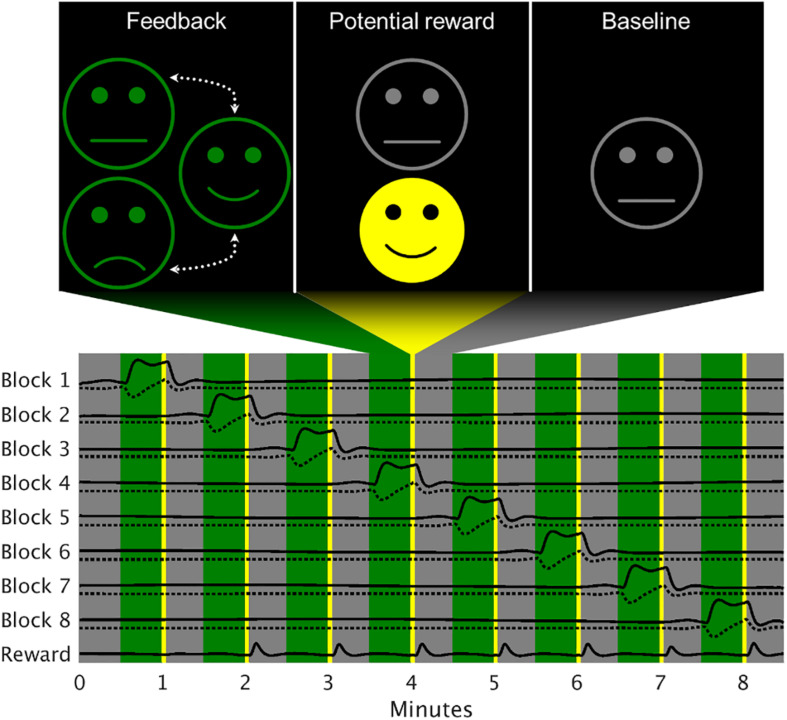
Exemplary model design matrix of a neurofeedback run. The feedback block activations were modeled separately (solid lines), each accompanied by a model for changes within the block (dotted line). Since the reward was of no specific interest for the analysis, a single regressor was used. Gray areas represent baseline periods, green areas the active regulation condition and the yellow stripes the time when the reward would be given (here seven out of eight times).

The NF runs consisted of eight active regulation blocks of 30 s flanked by baseline periods of the same duration. On the basis of the increased regulation success achieved using a smiling avatar as graphical representation of a cingulate target region’s activation (Mathiak et al., 2015), the feedback was displayed as the degree of smiling of a simple smiley face. The face was presented in gray with a neutral expression during the baseline and in green with a variable expression in the active regulation periods. For the PR-only group, the possible expressions ranged from neutral to strongly smiling in case of sufficient deactivation (Drevets et al., 2008; Hamilton et al., 2011), whereas the PR + PP group also received sad expressions if the regulation was going in the wrong direction (i.e., for activation). In other words, any negative feedback in the PR + PP group is represented as a neutral facial expression in the PR-only one. The maximally positive and negative expressions were thresholded at ± 5 percent signal change (PSC). As additional motivation (Chiew and Braver, 2011), indicator of successful strategies (Konicar et al., 2015) and for providing additional intermittent feedback (Smith and Kimball, 2010; Emmert et al., 2017; Hellrung et al., 2018) a yellow reward smiley with maximally positive expression was displayed directly after a regulation phase for 3 s if the median of the second half of that block showed at least −0.5 PSC (Figure 1). Besides the feedback, no further stimuli were presented to the subjects and the only instruction given was to make the face smile with any appropriate mental strategy. For self-motivation and in order to foster learning, the subjects were told to use their most successful strategy up to then during the second half of the last NF run each session. During the TRs, the green smiley remained neutral (did not change its expression due to feedback) and no indications of successful strategies were shown.

### Data Acquisition

The neuroimaging data were recorded using a Siemens Prisma 3T scanner (Siemens, Erlangen, Germany) equipped with a 64-channel head coil. For the FL, NF, and TR, a multiband-accelerated echo planar imaging sequence was optimized for high temporal resolution within the computational limits of TBV in order to provide sufficient data for individually modeling the single regulation blocks: echo/repetition time = 30/483 ms, multiband factor = 8, field of view = 190 × 190 × 140 mm at 76 × 76 × 56 voxels, yielding an isotropic resolution of 2.5 mm, flip angle = 46°, bandwidth = 2630 Hz/Px. The phase-encoding direction was set to posterior-anterior to avoid compression of the frontal cortex due to susceptibility artifacts.

The PANAS-SF and VAS data were acquired by means of paper-and-pencil questionnaires.

### Neuroimaging Data Processing

Online processing was conducted using TBV. Volumetric smoothing was set to 5 mm full width at half maximum (FWHM). No temporal averaging was performed. The feedback was presented using the Psychtoolbox (Brainard and Vision, 1997) and MATLAB (The MathWorks, Natick, MA, United States). As a reference, the median signal of the baseline preceding each regulation block was calculated (excluding the first 6 s to compensate for the hemodynamic delay), the feedback signal converted to PSC and thresholded at ± 5%.

Offline processing was conducted using Statistical Parametric Mapping, version 12 (SPM12), and the BrainWavelet Toolbox (Patel et al., 2014). The data was slice-timing corrected to the middle slice, realigned using Fourier interpolation in two passes to the first and afterward the mean image, which was subsequently used as normalization target to the standard space defined by the Montreal Neurological Institute (MNI) at the original isotropic resolution (Mueller et al., 2017). The images were further gray-matter-masked using a custom template based on the SPM and Harvard-Oxford tissue probability maps, and smoothed with a Gaussian kernel of 5 mm FWHM. The gray matter (GM) voxels were finally cleaned using wavelet despiking, where the “chain search” option was set to “harsh” in light of the high sampling rate.

### Temporal Modeling

For the fMRI whole-brain analysis, each regulation block was modeled individually using a boxcar and an orthogonalized sawtooth function in SPM12. The latter function was used to detect linear changes during the active condition, which correspond to the subjects’ ability to influence the sgACC within a block. A single regressor was also added for all rewards shown and orthogonalized to the single blocks. All model regressors were convolved with the canonical hemodynamic response function (HRF). An overview of the 1st-level model is presented in Figure 1. Nuisance regressors were defined via the Friston-24 model (Friston et al., 1996) and an adapted version of the CompCor approach (Behzadi et al., 2007), which individually derived the number of combined white matter and cerebrospinal fluid components via an automated scree method. Prior to component extraction, the tissue signals were subjected to the same wavelet despiking as the GM voxels and z-scored. No highpass filtering was applied to avoid interferences with the estimation of the non-periodic regressors and the autocorrelation method was set to “FAST” (Olszowy et al., 2019). The regulation block estimates were converted to PSC prior to further analysis.

For the behavioral analysis, the feedback signal was also modeled by extracting the above-described regressors from the SPM analysis [HRF-convolved and pre-whitened boxcar and sawtooth functions (normalized to an amplitude of 1) to compensate for the physiological delay]. In contrast to the feedback presentation, the baseline periods were not forced to zero and the regulation blocks not limited to positive values for the PR-only group for the GLM fitting. By design, the resulting regression coefficients were in the range [−1, 1] for the constant and [−2, 2] for the linear terms. The coefficients were Fisher-z-transformed to achieve an unbound distribution {after scaling the linear terms to [−1, 1]}.

### Statistical Modeling

For the sgACC analysis, the transformed *z*-values of the feedback time courses were entered into a linear mixed effects (LME) model in MATLAB with the factors Group (G: PR, PR + PP), Session (S: S1, S2) and Run (R: TR1, NF1…3, TR2), Block as single, linear regressor (B: B1…B8) with mean corrected to zero and a random intercept per subject. Additional models were estimated for the NF or TR data only to exclude the influence of the presence or absence of the visual feedback. In a first run, interaction effects with the group factor were investigated. After removal of the interaction terms, also the pure main effects of the factors were estimated. The factors were dummy-encoded and the first level of each factor (G: PR, S: S1, R: TR1 or NF1) and their interactions were always used as reference. All analyses were run for the boxcar and the sawtooth coefficients separately. Due to the orthogonality and, hence, independence of the model functions, a Sidak correction was applied to the two LME models of the target region and three separate datasets (i.e., six models). *Post hoc* comparisons for the runs were again corrected for multiple testing using the Sidak method (five for the combined NF + TR dataset and three for the NF data only).

The complementary whole-brain analysis used the Sandwich Estimator (SwE), version 2 (Guillaume et al., 2014), which allowed for inclusion of subjects with missing scans. The basic model comprised all available NF and TR blocks and was set up using the “Classic” SwE, “C2” small-sample adjustment and the “Naïve” degrees of freedom (DoF) estimation. The same models and analytical strategies were followed as for the feedback signals. The SwE results were corrected for false discovery rate (FDR) at voxel-level (*q* ≤ 0.025 for each side).

The PANAS-SF sum scores (positive minus negative items) and the VAS self-ratings were also analyzed using LME with G and S factors, a random intercept per subject and an additional factor for the pre/post-session assessment of the questionnaire. Since the VAS data was limited on [0, 100], it was also rescaled to [−1, 1] and z-transformed. Restricted maximum likelihood was used for all model fittings.

The averages over significant main effects of Run were Spearmen partially correlated (corrected for the two sessions) with the psychometric scores on an exploratory basis given the limited sample size. A possible relationship between the average and regulation trend was assessed for all blocks again using partial Spearman correlation with corrections for all factors.

## Results

### Demographics

Of the 13 volunteers enrolled in the study, one was excluded based on movement patterns locked to the NF time course and the self-report of having concentrated on his breath as a regulation strategy, potentially leading to a biased blood-oxygenation-level-dependent signal. The remaining 12 subject were included in the current analysis. Due to technical issues, for one subject, two of the NF runs of the second session were excluded and for another subject no regulation trials could be conducted in the second session. Detailed demographic information in given in Table 1.

**TABLE 1:** Group demographics.

Group	*N*	Age: Mean ± Std	Sex: m/f	Days between sessions: min/median/max
PR	6 (5)	24.50 ± 2.26 (24.80 ± 2.39)	3/3 (3/2)	1/2.5/13 (1/3/13)
PR + PP	6 (5)	27.67 ± 5.99 (26.20 ± 5.36)	4/2 (3/2)	2/3/11 (2/3/11)

*The values for only the subjects with complete neurofeedback runs are given in parentheses. RP, positive reinforcement; PP, positive punishment.*

### Psychometric Scores

**FIGURE 2:**
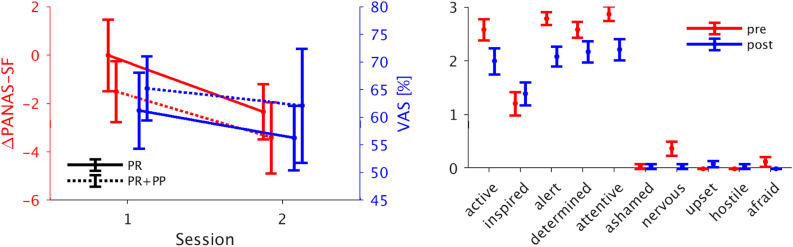
Psychometric scores. **Left panel:** Mean ± standard error of the PANAS-SF questionnaire post-pre difference and the VAS self-ratings for the separate groups. Both scales are lower for the second compared to the first session. **Right panel:** The single items of the PANAS-SF questionnaire for the pre- and post-measurement assessment (mean ± standard error). Four out of five positive items show a decrease after the NF training. PANAS-SF, Positive And Negative Affect Schedule – Short Form; VAS, visual analog scale.

The LME analysis of the VAS data yielded no significant interaction or main effects. Also no interaction effects were found in the PANAS-SF data but a significant main effect of the time of assessment {*p* < 0.05, β = −1.67, 95% confidence interval = [−2.97, −0.38]} with the scores being significantly lower after the NF sessions. The VAS and the sum scores of the PANAS-SF as well as its single items are presented in Figure 2.

### Regulation

During S1, the PR + PP group showed a significantly higher average regulation success then the PR-only group compared to S1. This is indicated by a significantly negative interaction effect for the PR + PP group in S2 (fitted mean PSCs: PR-only S1: −0.049, PR-only S2: −0.209, PR + PP S1: −0.277, PR + PP S2: −0.098). A significant effect of NF3 compared to the TR1 indicates a general learning effect over each session. However, the effect was not transferrable to the post-TRs (TR2).

**FIGURE 3:**
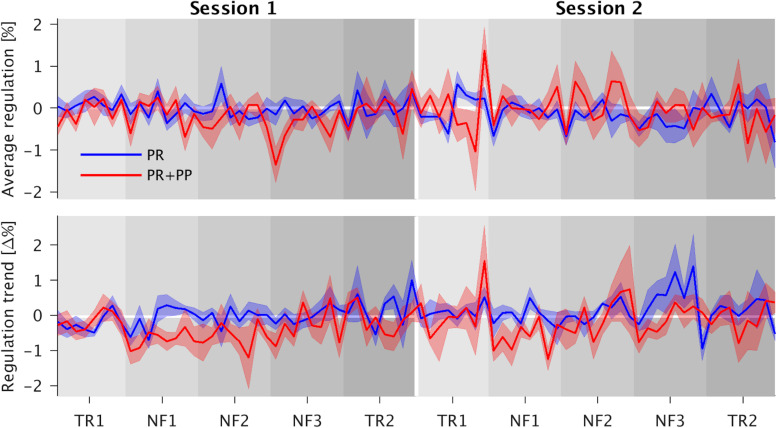
Time courses of the average regulation and regulation trends with their respective standard errors. In the top graph the interaction effect present for the three neurofeedback runs (NF1-3) can be roughly estimated as the positive reinforcement and positive punishment (PR + PP) group lies mostly above the PR-only group during the first session (left half) and below during the second one (right half). The increased trend in the PR + PP group as compared to the first transfer runs (TR1) as reference is more clearly visible in both sessions. The trend approaches zero over the duration of the residual sessions.

A significantly stronger regulation trend was found during NF1 of the PR + PP group compared to TR1 of the PR-only group (fitted mean PSC changes: PR-only TR1: −0.045, PR-only NF1: −0.027, PR + PP TR1: −0.041, PR + PP NF1: −0.585). For the combined NF and TR data as well as the NF dataset alone, significantly smaller regulation trends of the sgACC were found for S2 as well as a decreasing trend over the training blocks (B). In the overall data, a significantly stronger trend in the regulation was observed during NF1 compared to TR1. For the NF data alone, a further decrease in the regulation trend was found for the third (NF3) compared to the first (NF1) run. Additional *post hoc* comparisons confirmed these effects. The significant factors and covariates of the models and *post hoc* comparisons are listed in Table 2. The group-related interaction effects are further depicted in Figure 3.

**TABLE 2:** Significant linear mixed effects results and *post hoc* comparisons for the feedback region.

Model	Effect	Dataset	Factor/Covariate	β	95% CI	*p*-value	β*	PSC
Average	Interaction	NF	S2:PR + PP	–0.068	[−0.112, −0.023]	0.020	–0.067	0.337
Average	Main	NF + TR	NF3	0.047	[0.019, 0.075]	0.007	0.047	–0.234
Trend	Interaction	NF + TR	NF1:PR + PP	0.057	[0.022, 0.093]	0.010	0.114	–0.571
Trend	Main	NF + TR	S2	–0.019	[−0.031, −0.008]	0.007	–0.039	0.193
Trend	Main	NF + TR	NF1	0.025	[0.007, 0.043]	0.033	0.051	–0.253
Trend	Main	NF + TR	B	–0.005	[−0.008, −0.003]	<0.001	–0.011	0.055
Trend	Main	NF	S2	–0.024	[−0.039, −0.009]	0.008	–0.048	0.240
Trend	Main	NF	NF3	–0.029	[−0.046, −0.011]	0.008	–0.058	0.289
Trend	Main	NF	B	–0.007	[−0.01, −0.004]	<0.001	–0.013	0.066

**Model**	**Effect**	**Dataset**	**Comparison**	**β**	**95% CI**	***p*-value**	β*	**PSC**

Average	Main	NF + TR	NF3-TR1	0.047	[0.019, 0.075]	0.033	0.047	–0.234
Trend	Interaction	NF + TR	NF1:PR + PP-TR1:PR	0.057	[0.022, 0.093]	0.050	0.114	–0.571
Trend	Main	NF + TR	NF3-NF1	–0.029	[−0.047, −0.011]	0.042	–0.058	0.291
Trend	Main	NF + TR	TR2-NF1	–0.034	[−0.052, −0.016]	0.008	–0.067	0.337
Trend	Main	NF	NF3-NF1	–0.029	[−0.046, −0.011]	0.023	–0.058	0.289

The average was modeled using a boxcar and the trend within a regulation block with a sawtooth function. The first level of each factor was used for reference dummy coding for the basic models (Group: positive reinforcement only, Session: first session, Runs: first transfer run/first neurofeedback run). The references were swapper for the post hoc tests. Confidence intervals refer to the raw β estimates and were not corrected; β* denotes the back-transformed coefficients. All p-values were multiplicity-adjusted. CI, confidence interval; PSC, percent signal change; NF(1/3), (first/third) neurofeedback run; TR(1/2), (first/second) transfer run; S2, second session; PR, positive reinforcement; PP, positive punishment; B, blocks.

### Correlation With Psychometric Measures

The VAS and difference in PANAS-SF scores were correlated with the average regulation success of NF3 and the average trends over NF1 and NF3. The scores themselves showed a moderate correlation of *ρ* = 0.41. The VAS ratings positively correlated with the regulation average over NF3 with *ρ* = 0.45 and the trend over NF1 with *ρ* = 0.33. The post-pre difference of the PANAS-SF scores showed a higher correlation of *ρ* = 0.58 with the regulation success in NF3 and was the only value approaching significance (*p* = 0.061). All other coefficients were of small magnitude (|ρ| < 0.1). There was no significant correlation between the regulation successes and trends.

### Whole-Brain Analysis

**FIGURE 4:**
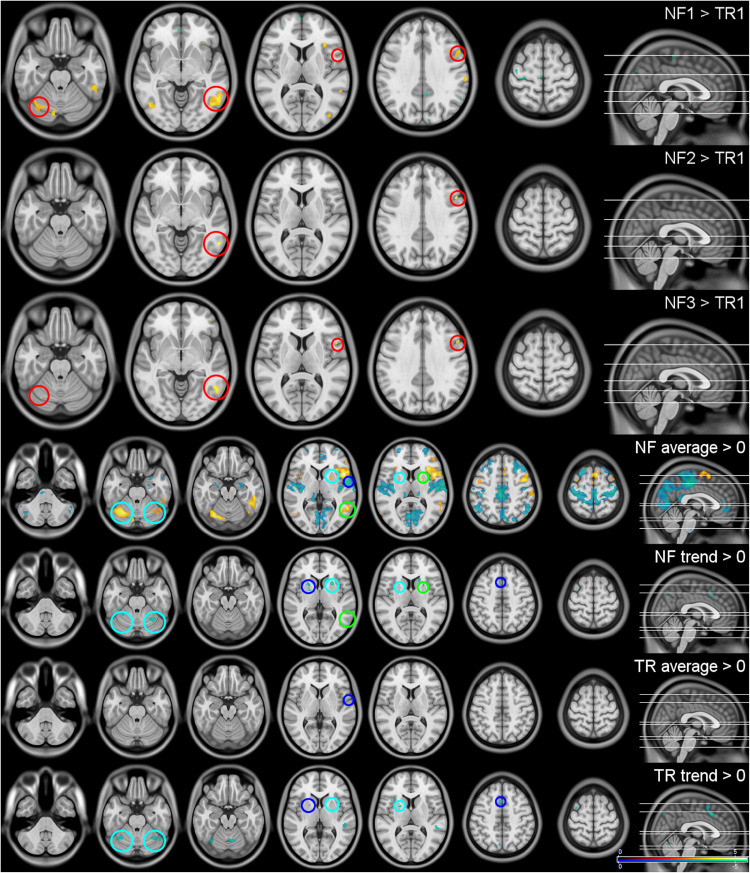
Whole-brain results. The first three rows show the activation differences between the neurofeedback runs (NF1-3) and the first transfer run (TR) as reference. As there were no significant differences in whole-brain activation within the three NF and the two TRs, they were summarized (TR/NF average) and are shown with their respective linear trends (TR/NF trend). All results were significant at a peak-level false discovery rate of *q* ≤ 0.05 two-sided. Red circles indicate regions significantly activated in multiple NF runs, blue circles effects visible in the NF and TRs, green circles regions that show activation as well as temporal trends within the same condition and cyan circles areas that display activations and trends across NF and TRs. The top three rows are shown at *z* = −24, −6, 9, 34, 63, the lower four at *z* = −41, −26, −20, 4, 9, 48, 64.

The most significant result, as well as the largest and any further significant cluster with an extent of 50 voxels or more are listed in Table 3. No significant interaction or linear trend effects were found for the comparison between the scans. The following results are based on the reduced models without interaction terms for the average activation per block (Figure 4, top three rows): Significant differences in brain activation were found between the single NF runs and the first TR as reference but not within the TR or NF runs. Stronger activations in the attention networks were especially prominent in NF1 and similar patterns, although to a lesser extent, in the remaining two NF runs (Figure 4, red circles).

**TABLE 3:** Details on selected significant whole-brain results.

Contrast	Coordinates	*q*-value	Cluster size	AAL region	Functional network	Neurosynth association
NF1 > TR1	53/13/30	0.013	106	Frontal inferior operculum	DA	Parietal, Posterior inferior
	−17/−72/−20	0.013	236	Cerebellum 6		VI
	48/−67/−10	0.013	496	Cerebellum crus 1	DA	Fusiform, Fusiform face
	66/−27/30	0.013	77	Supramarginal	VA	Pain, Limb
TR1 > NF1	−34/−14/65	0.013	45	Precentral	SM	Finger, Finger movements
NF2 > TR1	50/10/32	0.005	5	Precentral	DA	Task, ASD, parietal
	56/−60/−5	0.024	15	Temporal inferior	Da	Visual, vision
NF3 > TR1	50/10/30	0.017	43	Frontal inferior operculum	DA	Task, ASD
	48/−57/−12	0.024	77	Temporal inferior	DA	Occipito
NF avg. > 0	46/10/5	<0.001	1091	Insula	VA	Insula, Noxious
	−30/−67/−25	<0.001	387	Cerebellum 6		Cerebellum, VI
	3/13/58	0.001	377	Supplemental motor area	FP	Pre supplementary
	56/−57/2	0.001	739	Temporal mid	DA	Motion, Spectrum disorder
	58/−37/20	0.002	245	Temporal superior	VA	Action observation
	−47/−70/−5	0.002	98	Occipital inferior	Vi	Visual, ASD
	−42/−4/52	0.007	62	Precentral	DA	Premotor, Eye movements
NF avg. < 0	53/−4/0	0.002	942	Temporal superior	SM	Acoustic
	−4/−30/48	0.002	6375	Cingulum mid	SM	Foot
	30/23/52	0.003	487	Frontal mid	DM	Gyrus middle, Behavioral response
	46/−64/42	0.003	493	Angular	DM	Connectivity, SN
	−20/18/65	0.003	397	Frontal superior	FP	Discriminated
	−50/−54/50	0.003	271	Parietal inferior	FP	Parietal, Lobule IPL
	46/−64/−40	0.003	56	Cerebellum crus 2		
	−22/58/10	0.003	189	Frontal superior	FP	Social cognition, Frontopolar
	30/−22/−15	0.003	129	Parahippocampal	HI	Hippocampal, anterior Hippocampus
	6/43/0	0.003	185	Cingulum anterior	DM	Anterior cingulate, Taste
	−12/−30/8	0.004	61	Thalamus	BG	Thalamus, Cortex thalamus
	26/63/18	0.004	134	Frontal superior	DM	OCD
	−47/50/−10	0.005	66		FP	
	−27/−30/−12	0.006	76	Parahippocampal	HI	Hippocampus, lobe MTL
NF trend < 0	23/13/2	0.009	54	Putamen	BG	Putamen
	28/−57/−30	0.010	78	Cerebellum 6		Cerebellum, Rehearsal
TR avg. < 0	56/−2/5	0.002	6	Temporal superior	SM	
TR trend > 0	36/−27/18	0.006	1	Heschl	SM	Posterior insula
TR trend < 0	−4/16/42	0.004	141	Cingulum mid	VA	Task, Choose
	−40/−54/−32	0.004	156	Cerebellum crus 1		Cerebellar, VI
	40/−54/−35	0.004	147	Cerebellum crus 1		Cerebellar, VI
	8/−64/−22	0.007	91	Cerebellum 6		Cerebellum, Inferior superior
	−34/43/25	0.007	60	Frontal mid	VA	Dorsolateral, Conductance

*The most significant as well as the result contained in the largest cluster are shown for the comparison of the neurofeedback (NF) with the first transfer run (TR), and the average and linear trend summarized over all respective runs of the two conditions. Clusters with 50 or more voxels are additionally listed for all inferences. The respective region of the automatic anatomic labeling (AAL) atlas, the functional network from the Yeo atlas (Yeo et al., 2011) and the Neurosynth terms with the highest z-score and posterior probability are given for each coordinate if available. VI, visual; SM, somato-motor; DA, dorsal attention; VA, ventral attention; FT, fronto-temporal; FP, fronto-parietal; DM, default mode; HI, hippocampal; BG, basal ganglia.*

All NF and TRs were included in the assessment of the respective baseline activations, as intra-condition differences were found not to be significant (Figure 4, lower four rows): Activation during the regulation periods could be observed especially in the cerebellum, the supplementary motor area (SMA), the attention network (anterior insulae, parts of the temporal lobes) and the limbic system [anterior thalamus, putamen, caudate nucleus (CN)]. Deactivations were found in the somato-motor (SM), fronto-parietal (FP), and default mode (DM) networks, the bilateral (para-)hippocampi, posterior thalamus and the pons. A negative trend during the regulation was present mainly in the putamen, CN and cerebellum. The TRs showed similar but considerably weaker effects (blue and cyan circles in Figure 4).

The analysis of changes within the regulation blocks mostly identified negative activation trends in regions that showed increased activation especially during the NF runs (cerebellum, especially lobule 6, BG, and the attention networks during the TRs; green and cyan circles in Figure 4).

### Debriefing

The strategies reported as being most effective during the debriefing generally fall into two categories: positive autobiographical memories (PAM) or imagination of positively connoted situations. In each group, three of the six subjects described the (potential) PP as helpful, two as distracting or stressful and one as having no influence. Of note, three female volunteers (two in the PR + PP group) reported having achieved better regulation success with closed eyes (more rewards) and one male subject (in the PR + PP group) focused on a point outside of the smiley face. Lastly, several volunteers described the overall experience as positive and the feedback sessions as relaxing but also exhausting. The repetitive application of the same regulation strategy was also anecdotally mentioned as tiring.

## Discussion

The current study investigated the promotive potential of PP and its influence on the related but previously generally disregarded dynamics in NF learning.

### Psychometrics and Strategies

The decrease in PANAS-SF score, which was intended to capture effects of the NF training on emotions, can be explained by the demanding task but also subjectively relaxing scanner environment (as reported during the debriefing) in conjunction with the focus on activity of the positively valenced items. Similar NF-related decreases on the positive affect scale of the PANAS have been previously reported (Lorenzetti et al., 2018; Zahn et al., 2019). It has further been shown that NF has a significant influence on feelings of fatigue (Rana et al., 2016) which, in turn, might affect the regulation performance (Moll et al., 2014). Especially novelty and the related attention could play a major role (Moll et al., 2014), which is in line with activation in the dorsal and ventral attention networks and reports of the current population. The correlation with the individual regulation success (*ρ* = 0.58) still points toward a relationship between the psychometric measure and the NF training. Smaller positive correlations were found for the self-rating via the VAS, including the regulation trend. It might thus be speculated that the dynamics within each training block are partly reflected in the participants’ subjective impression of their success but not on a behavioral level. This, however, needs to be confirmed in a larger population since the correlations reported here should be understood as effect sizes in the light of the limited sample size.

Regarding the participants who reported achieving higher regulation success when not looking at the smiley, analogies to continuous vs. intermittent feedback (Emmert et al., 2017; Hellrung et al., 2018) and “operant conditioning” vs. “delay-retention” (Renner, 1964; Kulhavy and Anderson, 1972; Smith and Kimball, 2010) seem reasonable. Since NF-based as well as learning-theoretical investigations are inconclusive on which is the more effective feedback scheme, a combination of both was employed in the current design.

### The Role of Positive Punishment

With the PR + PP group having a higher regulation success during S1 and stronger regulation trends (i.e., more control in the desired direction) in NF1, this finding in particular indicates potentially faster learning when PP is added as a feedback mechanism. Beyond these group-related effects, a generally higher trend was observed in NF1 compared to TR1 and a significantly lower trend in NF3 compared to NF1. In conjunction with the significant average regulation in NF3, it can be speculated that an increased regulation trend represents faster initial learning which reduces as successful strategies are identified. This would be corroborated by the observation of a significant decrease in the trends within single runs. The lack of a direct correlation between the regulation average and the trend is interpreted as subjects having gained control over the target region from different starting points (i.e., corrected the direction of the activation as well as improved it).

PR was previously shown to provide a more encouraging EEG feedback mechanism than negative reinforcement and also generated more positive affect (Reinschluessel and Mandryk, 2016). In a study using EEG NF for children with learning disabilities, it was further reported that PP alone led to additional improvements in the understanding of reading and reasoning as well as greater EEG-related changes compared to using PR (Fernández et al., 2008). It is possible that negative and positive feedback (i.e., positive reinforcement and punishment) work via different neuronal mechanisms with varying influences on emotion, motivation, and learning success (Chiew and Braver, 2011). This idea is of particular interest in scenarios such as the current one, where feedback and reward cannot be clearly distinguished since the feedback itself was provided by means of a social reward – a smile (Cordes et al., 2015; Mathiak et al., 2015). That there was still no significant activation difference between the groups on the whole-brain level is likely due to the similarities in the goal of the training and the applied strategies. Even though reward-based and avoidance (of punishment) learning were shown to correspond to different neuronal activation patterns (Kim et al., 2015), the amount of PP could and should by design be implicitly reduced in this study making it only a transient condition. Larger sample sizes might, however, uncover subtle activation differences in the target and related regions and varying relationships with the training results as it was shown in Argyelan et al. (2018) where learning positively correlated with the response of the putamen to punishment but not reward.

From the perspective of decision making, the additional punishment can be seen as a cost of learning that ought to be minimized. Within this context, the respective costs have been identified as a better model for choices than the expected reward (Gray and Tallman, 1987), which would partly explain the supportive aspects or even superiority of PP over PR (Fernández et al., 2008). However, contrary to previous investigations, PP was here not used as alternative feedback mechanism but as an additional one, doubling the feedback range. Thus, not only complementary feedback was available but also more information on the effectiveness of the current regulation strategy, which probably also contributed to the faster learning. Future studies aiming at investigating the influence of the information content alone might hence need to scale the feedback accordingly.

More complex underlying phenomena may also be indicated by the fact that children regained control over their brain slow cortical potentials after the feedback direction was switched – of what they were left unaware – without changing their control strategies (Siniatchkin et al., 2000). In line with Siniatchkin et al. (2000), it might be hypothesized that the presentation of the feedback plays a more important role than the search for an effective regulation strategy. In another study supporting this reasoning fMRI feedback was provided to the subjects via positively (PR) or negatively (PP) connoted auditory stimuli without informing them that the sounds depend on their brain activation (Ramot et al., 2016). The majority of subjects showed a modulation of the target regions in the intended direction and related changes in functional connectivity without awareness of the NF training. It would of course be interesting to examine whether this result could also be achieved in the absence of PP.

### Emotion Regulation and Dynamics

The putamen, CN and lobule 6 of the cerebellum all showed activation during the regulation periods accompanied by a decrease over time. For the CN, involvement in learning from prediction errors had been shown (Schiffer et al., 2012). This adds to the previous argumentation for a model of PP-driven cost reduction (Gray and Tallman, 1987). In case of an application to psychiatric populations and possible concomitant medication, modulatory effects should further be considered (Graf et al., 2011). An involvement in emotion processing of the CN and the putamen was also found for subjects implicitly reading neutral or unpleasant words (Szekely et al., 2017). Moreover, the putamen was shown to play a role in volitional emotion control (Seo et al., 2014). A stronger activation in the preparatory compared to the regulatory phase could also explain the negative trend visible in the current results. Beyond this, the CN and the putamen are known to be involved in learning and memory (Packard and Knowlton, 2002), essential aspects of NF training. Furthermore, potential interactions of the emotional and cognitive facets of the task would be of particular interest (Borchardt et al., 2017). For the cerebellum, a functional organization comparable to that of the cerebrum was suggested (Stoodley and Schmahmann, 2010, 2018) where lobule 6 is involved in cognitive processes and especially mental imagery (Higuchi et al., 2007). Furthermore, contributions to working memory and motor learning have been shown (Bernard and Seidler, 2013; Kowalczyk et al., 2020), which are also reasonable in the context of NF given the necessity to learn and remember strategies, often including the imagination of physical activities. Specific activity in other regions of the cerebellum was also reported in NF before (Banca et al., 2015).

### Limitations

The major limitation of the current study is the sample size which renders investigations besides the main hypothesis exploratory. This is particularly true for the correlations with the psychometric data, which were therefore primarily interpreted as effect sizes. No sham group was included since the focus of this study was to investigate the influence of additional PP compared to a standard feedback scheme. This allows for interpretation of the effect of PP when added to PR, but not PR alone. The subject- and session-specific delineation of the sgACC served the individualization of the NF training but could likely increase residual differences in the activation pattern after spatial normalization explaining a certain discrepancy between the ROI and whole-brain analysis. Even though the subjects gained volitional control over the region, this did not generalize to the TRs. This finding is, however, in line with the only previous study using the sgACC as NF target (Hamilton et al., 2011). On the contrary, the amygdala, for which transferability of the regulation was shown multiple times, also exhibits decreased activation to subsequent aversive stimuli without applying a regulation strategy (Walter et al., 2009). Such divergent findings for functionally related brain regions demand further investigation. A final aspect that should be considered in future studies is a potential non-linear effect on emotion-related training regions when emotionally connoted feedback is used.

## Conclusion

The current work investigated whether additional positive punishment facilitates emotion regulation learning by broadening the available range of the feedback and providing a complementary training mechanism. In contrast to conventional analyses, this was realized by separately modeling the average of and trends in the regulation signals of each single feedback period, allowing temporal effects within blocks, runs, sessions, and over the study to be assessed. Additional positive punishment was shown to lead to a higher regulation success in the first session and increased controllability in the desired direction during the respective first runs, both indicating faster initial learning. It therefore seems that the reduction of errors also in neurofeedback represents an important driving factor of learning and complements the reward spectrum to facilitate self-control over brain activity. Future work should aim for a more detailed investigation of different feedback types and target regions to address the generalizability of findings, differences and advantages of each brain area.

## Data Availability Statement

The raw data supporting the conclusions of this article will be made available by the authors, without undue reservation, to any qualified researcher.

## Ethics Statement

The studies involving human participants were reviewed and approved by the ethics committee of the Medical University of Vienna. The participants provided their written informed consent to participate in this study.

## Author Contributions

MK, AH, and RL planned the study. MK recorded and analyzed the data under the methodological supervision of AH and SR. PM and GG performed the pre-screenings and final exams. PM provided the medical support throughout the study. RL was the principal investigator and supervisor of the study. All authors have read and revised the manuscript and agreed to the final version.

## Conflict of Interest

RL received travel grants and/or conference speaker honoraria within the last 3 years from Bruker BioSpin MR, Heel, and support from Siemens Healthcare regarding clinical research using PET/MR. He is a shareholder of BM Health GmbH since 2019. Preliminary analyses of the data were presented at the annual meeting of the Organization for Human Brain Mapping 2020. The remaining authors declare that the research was conducted in the absence of any commercial or financial relationships that could be construed as a potential conflict of interest.
